# Comparative Stress Analysis of Maxillary Implant Prostheses With Different Retention Designs and Restorative Materials

**DOI:** 10.7759/cureus.103949

**Published:** 2026-02-20

**Authors:** Ashutosh R Singh, Sandeep Kumar, Rajnish Aggarwal, Rahul Sharma, Aniruddha Deokate, Harjot Kaur, Ibadat K Sra

**Affiliations:** 1 Department of Prosthodontics, Surendera Dental College and Research Institute, Sri Ganganagar, IND; 2 Department of Oral and Maxillofacial Surgery, Dr. S.S. Tantia Dental College, Hospital and Research Centre, Sri Ganganagar, IND; 3 Department of Prosthodontics, Mittal Dental Care Implant and Maxillofacial Clinic, Sangrur, IND

**Keywords:** cement, finite element analysis, implant, prostheses, screw, stresses, zirconia

## Abstract

Introduction: Implant-supported restorations are widely used to replace missing teeth; however, their long-term success depends largely on the biomechanical behavior of the implant-prosthesis complex. This study aimed to evaluate and compare stress distribution patterns in maxillary implant-supported restorations with different retention methods and crown materials under axial and oblique loading conditions using three-dimensional finite element analysis.

Materials and methods: A three-dimensional finite element model of a partially edentulous maxillary premolar region was constructed. A titanium dental implant with an abutment was positioned vertically within the cortical and cancellous bone. Four restorative configurations were analyzed: cement-retained porcelain-fused-to-metal, screw-retained porcelain-fused-to-metal, cement-retained zirconia, and screw-retained zirconia crowns. All materials were assumed to be homogeneous, isotropic, and linearly elastic. Two loading conditions were simulated: a vertical axial load of 100 N applied along the implant axis and an oblique load of 100 N applied at 30° to the implant axis. von Mises stress distribution was evaluated in the crown, abutment, implant, prosthetic screw, and surrounding bone.

Results: Cement-retained restorations demonstrated a more uniform stress distribution than screw-retained restorations, particularly at the implant-abutment interface. Porcelain-fused-to-metal crowns exhibited relatively lower stress concentrations in prosthetic components than zirconia crowns. Oblique loading generated higher stress values than axial loading in all models, with maximum stresses localized at the implant neck and the crestal cortical bone.

Conclusion: Within the limitations of this finite element study, cement-retained porcelain-fused-to-metal restorations exhibited more favorable biomechanical behavior in the maxilla than screw-retained titanium-based restorations. The results highlight the importance of retention type, restorative material, and occlusal loading direction in achieving optimal implant-supported restoration performance.

## Introduction

Implant-supported prosthetic rehabilitation has become a routine and predictable treatment option for replacing missing teeth. In addition to achieving satisfactory esthetics, these restorations must withstand functional loads and transmit them in a manner that preserves the integrity of the implant components and surrounding bone [1]. The biomechanical environment of the maxilla differs considerably from that of the mandible because of variations in bone density, cortical thickness, and trabecular architecture [2]. These anatomical differences may influence the distribution of occlusal forces around implants and therefore warrant independent evaluation.

One of the key clinical considerations in implant prosthodontics is the method of prosthesis retention. Screw-retained restorations offer advantages such as retrievability and simplified maintenance, whereas cement-retained prostheses are often preferred for their improved esthetics and passive fit [3]. Despite their widespread use, the biomechanical implications of these retention systems, particularly in the maxilla, remain a subject of ongoing debate.

The choice of restorative material is another factor that can affect load transmission. Metal-ceramic restorations have a long history of clinical success owing to their favorable mechanical behavior, whereas zirconia-based restorations are increasingly selected for their superior esthetic qualities and high strength [4,5]. However, differences in stiffness between these materials may alter the magnitude and distribution of stresses within the implant assembly and adjacent bone, particularly under non-axial loading conditions.

Three-dimensional finite element analysis provides a reliable and noninvasive method for evaluating stress patterns in complex implant-supported systems [6]. This three-dimensional finite element study aimed to evaluate and compare stress distribution patterns in maxillary implant-supported restorations with different retention methods and crown materials under axial and oblique loading conditions. The objectives of this study were to analyze stress transmission in cement-retained and screw-retained prosthetic designs, compare the biomechanical behavior of porcelain-fused-to-metal and zirconia crowns in the maxilla, and assess the influence of axial and oblique occlusal forces on stress distribution within the crown, abutment, implant, prosthetic screw, and surrounding cortical and cancellous bone.

## Materials and methods

This three-dimensional finite element study was conducted at the Department of Prosthodontics, Surendera Dental College and Research Institute, Sri Ganganagar, India. A geometric model representing a partially edentulous maxillary segment in the premolar region was constructed. The model consisted of an outer cortical bone layer surrounding a cancellous bone core, simulating bone characteristics typically found in the maxilla. The cortical layer was modeled with a uniform thickness, and the bone segment was smoothed to eliminate sharp contours that could artificially concentrate stress.

A commercially pure titanium screw-type dental implant with a diameter of 4.2 mm and a length of 13 mm was designed and positioned vertically within the maxillary bone model. The implant design incorporated a threaded body, a collar, and a rounded apex. A standard prefabricated abutment with appropriate dimensions and occlusal taper was connected to the implant to support the prosthetic crown.

Four different restorative configurations were created for comparative analysis: a cement-retained porcelain-fused-to-metal crown, a screw-retained porcelain-fused-to-metal crown, a cement-retained zirconia crown, and a screw-retained zirconia crown. All crowns were designed with identical anatomical contours and dimensions to ensure consistency among the models. The screw-retained designs included a prosthetic screw and access channel, whereas the cement-retained restorations incorporated a uniform cement layer between the crown and the abutment.

Three-dimensional models of the maxillary bone, implant, abutment, prosthetic screw, and crowns were developed using computer-aided design software and subsequently imported into finite element analysis software. The assembled models were discretized into tetrahedral elements. A finer mesh was applied in regions expected to experience higher stress, such as the implant-bone interface, implant neck, and abutment connection, whereas a coarser mesh was used in less critical areas to optimize computational efficiency.

All materials were assumed to be homogeneous, isotropic, and linearly elastic. The mechanical properties of cortical bone, cancellous bone, titanium, porcelain-fused-to-metal, zirconia, and the cement layer were assigned according to values reported in previous studies (Table 1) [7,8].

**Table 1:** Material properties used in the study

Components	Modulus of elasticity (GPa)	Poisson’s ratio
Cancellous bone	1.37	0.30
Cortical bone	13.7	0.30
Titanium	110	0.33
Zirconia	210	0.30
Metal alloy	220	0.35
Feldspathic porcelain	48.7	0.23

Boundary conditions were applied by constraining the outer surfaces of the maxillary bone model to prevent rigid-body motion. Two loading scenarios were simulated to represent functional occlusal forces. A vertical load of 100 N was applied along the long axis of the implant, and an oblique load of 100 N was applied at 30° to the implant axis on the occlusal surface of the crown [9].

Finite element calculations were performed to determine the von Mises stress values in the crown, abutment, implant, prosthetic screw, and surrounding cortical and cancellous bone. The stress distribution patterns obtained from each model under both loading conditions were compared to evaluate the influence of retention type, restorative material, and load direction on the biomechanical performance of maxillary implant-supported restorations.

## Results

The three-dimensional finite element analysis revealed noticeable differences in stress distribution among the four restorative configurations under both axial and oblique loading conditions. Stress patterns were evaluated in the crown, abutment, implant, prosthetic screw, and surrounding cortical and cancellous bone using the von Mises stress criterion.

Across all models, the highest stress concentrations were consistently observed in the crestal cortical bone around the implant neck, whereas comparatively lower stress values were noted in the cancellous bone. This pattern remained similar regardless of the retention type or restorative material. Under axial loading, the stress distribution was relatively uniform along the long axis of the implant in all models. Cement-retained restorations exhibited more favorable stress patterns, with reduced stress concentrations at the implant-abutment interface and within the prosthetic screw region. In the cement-retained porcelain-fused-to-metal model, the stresses were more evenly distributed across the crown and abutment, with minimal transfer to the surrounding bone. The cement-retained zirconia model showed a higher stress concentration within the crown structure owing to its higher stiffness, although stress transfer to the implant components remained comparatively controlled (Table 2).

**Table 2:** von Mises stress in different structures under axial loading in cement-retained restorations

Structures	Zirconia (MPa)	Porcelain-fused-to-metal (MPa)
Crown	28.345	25.157
Implant	23.035	20.29
Abutment	37.494	32.663
Screw	8.561	7.487
Cancellous bone	4.064	3.697
Cortical bone	9.456	8.416

**Figure 1:**
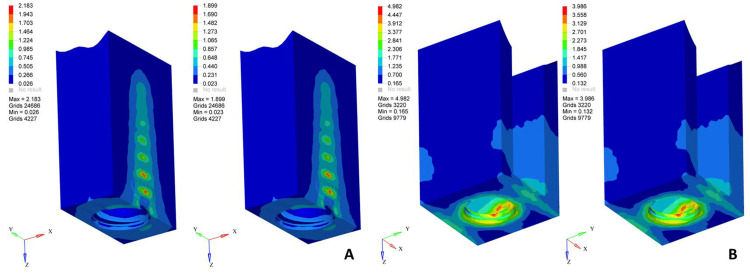
von Mises stress distribution patterns in screw-retained restorations under axial loading (left: zirconia, right: porcelain-fused-to-metal) on (A) cancellous bone and (B) cortical bone A vertical axial load of 100 N was applied along the long axis of the implant. The color scale represents von Mises stress values in MPa, where blue indicates minimum stress and red indicates maximum stress concentration. Original finite element analysis images of the simulated models.

In screw-retained restorations under axial loading, higher stress values were observed at the prosthetic screw and implant-abutment interfaces. The screw-retained zirconia model demonstrated greater stress concentrations in the prosthetic screw and abutment than the screw-retained porcelain-fused-to-metal model. The cortical bone around the implant neck exhibited slightly higher stress in the screw-retained configurations than in the cement-retained designs (Table 3) (Figure 1).

**Table 3:** von Mises stress in different structures under axial loading in screw-retained restorations

Structures	Zirconia (MPa)	Porcelain-fused-to-metal (MPa)
Crown and abutment	33.482	30.044
Implant	11.399	10.031
Screw	9.354	8.014
Cancellous bone	2.183	1.899
Cortical bone	4.982	3.986

Oblique loading produced a significant increase in stress magnitude in all models compared with axial loading. Maximum stress concentrations were localized on the loading side of the implant neck and the crestal cortical bone. Cement-retained restorations under oblique loading showed increased stress at the crown and abutment regions, with the zirconia model demonstrating higher stress accumulation within the crown structure (Table 4).

**Table 4:** von Mises stress in different structures under oblique loading in cement-retained restorations

Structures	Zirconia (MPa)	Porcelain-fused-to-metal (MPa)
Crown	63.160	36.767
Implant	97.399	85.771
Abutment	153.802	133.905
Screw	25.212	22.009
Cancellous bone	5.244	4.887
Cortical bone	31.553	28.062

**Figure 2:**
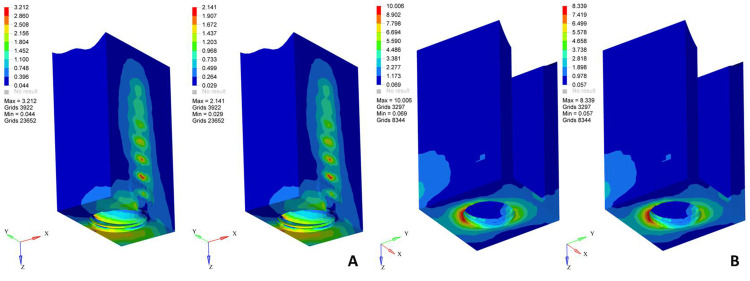
von Mises stress distribution patterns in screw-retained restorations under oblique loading (left: zirconia; right: porcelain-fused-to-metal) on (A) cancellous bone and (B) cortical bone An oblique load of 100 N was applied at a 30° angle to the long axis of the implant on the occlusal surface. The color scale represents von Mises stress values in MPa, where blue indicates minimum stress and red indicates maximum stress concentration. Original finite element analysis images of the simulated models.

Screw-retained restorations under oblique loading exhibited the highest overall stress values among all configurations. A pronounced stress concentration was observed in the prosthetic screw, implant-abutment junction, and cortical bone on the loading side (Figure 2). The screw-retained zirconia model showed the greatest stress concentration in the prosthetic components, whereas the screw-retained porcelain-fused-to-metal model demonstrated comparatively lower stress within the screw but increased stress transfer to the implant body (Table 5).

**Table 5:** von Mises stress in different structures under oblique loading in screw-retained restorations

Structures	Zirconia (MPa)	Porcelain-fused-to-metal (MPa)
Crown and abutment	97.340	83.728
Implant	50.543	44.587
Screw	30.613	26.059
Cancellous bone	3.212	2.141
Cortical bone	10.006	8.339

Overall, cement-retained restorations displayed more uniform stress distribution patterns, whereas screw-retained designs showed localized stress concentrations, particularly at the implant-abutment interface and prosthetic screw. Among the restorative materials, porcelain-fused-to-metal crowns demonstrated relatively lower stress transfer to the implant components and surrounding bone than zirconia crowns. Oblique loading generated higher stress levels than axial loading in all models.

## Discussion

The biomechanical performance of implant-supported restorations is a critical determinant of their long-term success. The proper transmission of occlusal forces from the prosthesis to the implant and surrounding bone is essential to minimize biological and mechanical complications [10]. The present three-dimensional finite element study evaluated the influence of retention method, restorative material, and loading direction on stress distribution in maxillary implant-supported restorations.

In the present study, the highest stress concentrations were consistently observed in the crestal cortical bone surrounding the implant neck, irrespective of the restorative configuration. This finding is in agreement with previous finite element studies, which have demonstrated that cortical bone bears the majority of occlusal loads owing to its higher modulus of elasticity than that of cancellous bone. The crestal region of cortical bone is particularly susceptible to stress concentration, making it a critical area for potential bone resorption and marginal bone loss [11,12].

Under axial loading, all models exhibited a relatively uniform stress distribution along the long axis of the implant. Axial forces are generally considered more favorable because they produce compressive stresses that are better tolerated at the implant-bone interface [13]. Cement-retained restorations demonstrated more uniform stress patterns and reduced stress concentrations at the implant-abutment interface and prosthetic screw region than screw-retained designs. This may be attributed to the absence of a screw access channel and the presence of a continuous crown-abutment interface, which allows for more even load transfer.

Although the present study demonstrated more favorable stress distribution in cement-retained restorations and porcelain-fused-to-metal crowns, these findings are not entirely consistent with the results reported by Lemos et al., who observed different stress patterns among retention systems and restorative materials [4]. The discrepancy between the studies may be attributed to differences in model design, implant-abutment connection geometry, bone quality simulation, and loading conditions. The present study focused on a maxillary bone model, which typically exhibits lower density and different trabecular architecture than mandibular bone, potentially altering stress transmission patterns.

Screw-retained restorations showed increased stress concentration in the prosthetic screw and implant-abutment junction, particularly under oblique loading. The presence of a screw access channel may act as a stress concentrator, leading to higher localized stresses in the screw and abutment components of the implant. This could increase the risk of mechanical complications, such as screw loosening or fracture, especially under non-axial forces.

Oblique loading produced significantly higher stress values across all models than axial loading. This observation supports the widely accepted concept that non-axial forces generate bending moments, which are more detrimental to the implant-bone interface [13,14]. The highest stress concentrations under oblique loading were observed at the implant neck and crestal cortical bone, indicating the importance of proper occlusal design to minimize lateral forces.

The type of restorative material also influenced stress distribution patterns. Zirconia crowns exhibited higher stress concentrations within the crown structure owing to their high modulus of elasticity. This increased stiffness limits deformation and results in greater stress accumulation within the restoration and underlying components. In contrast, porcelain-fused-to-metal restorations demonstrated a more favorable stress distribution, with relatively lower stress transfer to the prosthetic screw and implant components [5,15]. The slightly more flexible nature of metal-ceramic restorations may allow for partial absorption and dissipation of occlusal forces.

From a clinical perspective, the results of this study suggest that cement-retained restorations may provide biomechanical advantages, particularly in the maxilla, where bone quality is generally less dense than in the mandible. Reduced stress concentration at the implant-abutment interface may lower the risk of mechanical complications and marginal bone loss. Porcelain-fused-to-metal crowns demonstrated a more favorable stress distribution under both loading conditions, which may contribute to improved long-term stability of implant-supported restorations. However, zirconia crowns may still be preferred in situations where superior esthetics are required, provided that occlusal forces are carefully controlled and proper implant positioning is achieved. These findings emphasize the importance of minimizing oblique forces in implant-supported restorations. Proper implant angulation, occlusal scheme design, and careful adjustment of occlusal contacts are essential for reducing lateral loads and preventing excessive stress concentration in the crestal bone.

Despite the valuable insights provided by this study, certain limitations must be considered. The finite element models assumed that all materials were homogeneous, isotropic, and linearly elastic, which does not fully represent the complex anisotropic nature of biological tissues. The study also simulated static loading conditions, whereas intraoral forces are dynamic and vary in magnitude, direction, and frequency during function. Additionally, the models did not account for the presence of periodontal ligaments, bone remodeling, or the effects of long-term fatigue loading. Patient-specific anatomical variations and clinical factors, such as implant angulation, bone quality, and prosthetic fit, were not simulated.

## Conclusions

Within the limitations of this three-dimensional finite element study, cement-retained restorations demonstrated a more uniform stress distribution than screw-retained restorations in maxillary implant-supported prostheses. Porcelain-fused-to-metal crowns exhibited relatively lower stress concentrations within the prosthetic components and surrounding bone than zirconia crowns, particularly under oblique loading. Oblique forces produced significantly higher stress levels than axial loading across all models, with peak stresses consistently observed in the crestal cortical bone around the implant neck. These findings suggest that the careful selection of retention method, restorative material, and occlusal design is essential to achieve favorable biomechanical performance and enhance the long-term success of implant-supported restorations.
